# The intergenerational and transgenerational effects of miRNAs in fertility, obesity and stress responses

**DOI:** 10.1093/eep/dvag035

**Published:** 2026-10-26

**Authors:** Yiu Ching Tang, Kin Hei Lei, Emily Sze Wan Wong, Yi Yang, Keng Po Lai

**Affiliations:** Department of Applied Science, School of Science and Technology, Hong Kong Metropolitan University, Hong Kong SAR, China; Department of Applied Science, School of Science and Technology, Hong Kong Metropolitan University, Hong Kong SAR, China; Department of Applied Science, School of Science and Technology, Hong Kong Metropolitan University, Hong Kong SAR, China; State Key Laboratory of Marine Environmental Health, City University of Hong Kong, Hong Kong SAR, China; Department of Infectious Diseases and Public Health, Jockey Club College of Veterinary Medicine and Life Sciences, City University of Hong Kong, Hong Kong SAR, China; Department of Applied Science, School of Science and Technology, Hong Kong Metropolitan University, Hong Kong SAR, China; State Key Laboratory of Marine Environmental Health, City University of Hong Kong, Hong Kong SAR, China

**Keywords:** trans-generation, miRNAs, reproduction, obesity, mental health, stress-related

## Abstract

MicroRNAs (miRNAs) are key post-transcriptional regulators that play pivotal roles in diverse physiological and pathological processes. They primarily act by inhibiting protein translation and promoting degradation of messenger RNA (mRNA), thereby inducing post-transcriptional regulatory effects. Most miRNAs can travel through blood, in extracellular vesicles, and between maternal and fetal tissues. When altered miRNAs were present in germ cells, this induces epigenetic inheritance, which influences embryonic development after fertilization. Aberrant miRNA expression is associated with a wide range of human diseases. This review article will summarize current evidence on how miRNA dysregulation contributes to transgenerational effects on reproduction, metabolism, and stress-related phenotypes. This research focuses on the impacts of miRNAs on reproductive impairment, obesity, and stress-related phenotypes, and highlights evidence that these epigenetic changes can persist across multiple generations. Relevant studies were identified mainly through PubMed database using “miRNA” and “transgenerational effect”, with a third keyword according to the topic of interest, including “reproduction”, “hormones”, “spermatogenesis”, “obesity”, “high-fat diet”, “diabetes”, “stress”, and “therapy”. In reproduction, environmental pollutants (including endocrine disruptors) can alter miRNA expression, impairing gonadal development, reducing primordial germ cells, inducing granulosa cell apoptosis, and disrupting sex hormone synthesis. miRNA-dependent pathways also affect spermatogenesis, sperm function, and oocyte development. In obesity, miRNAs regulate adipogenesis by modulating signaling pathways and transcription programs that control fat-cell formation. Paternal high-fat diet and gestational diabetes can shift miRNA profiles, raising offspring risk for obesity and metabolic disease. Chronic stress changes miRNA expression in germ cells and embryos, shaping stress reactivity and social behavior in later generations. Prenatal maternal stress is linked to lasting psychiatric vulnerability in offspring, with effects potentially persisting across multiple generations without continued stress. Overall, miRNA dysregulation can impair reproduction, metabolism, and stress responses, and these effects may be transmitted transgenerationally via epigenetic mechanisms.

## Introduction

miRNA is an endogenous small RNA that regulates gene expression through an epigenetic mechanism, which involves gene expression alterations without changing the primary DNA base sequence. MiRNA was first discovered by the Ambros and Ruvkun groups in Caenorhabditis elegans in 1993 [1]. The first miRNA, lin-4, had a complementary sequence to that of the 3’ untranslated region of the gene lin-14. It was proposed that lin-4 regulates lin-14 at the post-transcriptional level through preventing lin-14 protein production. Lin-4 worked by blocking translation without reducing mRNA levels, emerging as a groundbreaking scientific discovery [1]. Soon after, another miRNA let-7, was found in many species, including humans, proving miRNAs were conserved evolutionarily [2]. Since then, miRNAs have been detected in various animal model systems, and their roles in gene regulation are well recognized [1]. MiRNAs are crucial for a variety of biological processes. Aberrant expression of miRNAs is associated with different human diseases. Extracellular miRNAs have also been discovered as potential biomarkers for diseases. Ying, Chang & Lin (2008) described miRNA as being usually about 18–25 nucleotides long, modulates other protein-coding genes, and pairs with mRNA to suppress protein production by blocking of translation and promoting degradation of the mRNA [3]. As it is usually partially complementary to different mRNAs, which results in imperfect base-pairing, one miRNA could regulate many different mRNAs. Lam et al. (2015) explained that the significance of miRNA lies in gene silencing to enable functional studies, works as a potential tool for silencing disease-related genes, and investigating gene function and regulation [4].

Epigenetics refers to the study of changes in gene function that are mitotically or meiotically heritable and that do not entail a change in DNA sequence [5]. In mammals, developmental programming is suggested to occur through epigenetic mechanisms, which involve DNA methylation, histone protein modifications, and miRNA regulation, where miRNA regulation would be the focus of this paper. MiRNA alterations could be transmitted through mitotic cell division, exposure to stimuli of the developing germ cells inside the placenta, or miRNA changes that are inherited through fertilizations between altered sperms and oocytes. The stimuli could be environmental cues, lifestyle problems, or stress-related phenotypes, changing the regulation of miRNAs. MiRNA marks that could be transferred from parents to the first progeny are regarded as intergenerational inheritance. This is best explained by the phenomenon of developmental programming induced by the intrauterine environment [6]. Developmental programming that could persist in unexposed progeny is regarded as transgenerational inheritance, meaning that the later generations are never directly exposed to the original stimulus [6]. Paternal transgenerational inheritance could be explained by the situation that the germ cells of F1 is exposed to stimuli, which undergoes spermatogonia differentiation and spermatogenesis to pass the miRNA alterations to the future F2 or even F3 generations. For maternal transgenerational inheritance, mitosis from primordial germ cells to oogonia and meiosis of oogonia to primary oocytes would transmit the miRNA patterns to the unexposed F2 and F3 progeny.

## Methodology

This review paper was conducted using a systematic approach to summarize the existing literature on how miRNA can cause transgenerational effects. A comprehensive literature search was performed in August 2026, with Pubmed being used as the electronic database and no restriction of publication year (Supplementary Fig. 1). Although transgenerational effects may be mediated through DNA methylation, histone modifications, and non-coding RNA regulation, such as miRNA, this review was restricted to miRNA-related mechanisms. Studies were used in the paper, only if they investigated the role, expression, regulation, or biological effects of miRNA in transgenerational outcomes. This study focuses on the transgenerational effect on reproductive systems, obesity, and mental health issues. For the keywords, the terms “miRNA” and “transgenerational effect” were used as fixed search terms, with an additional keyword based on the topic of interest. The topic-specific terms included “reproduction”, “hormones”, “spermatogenesis”, “obesity”, “high-fat diet”, “diabetes”, “stress”, and “therapy”.

Study selection used a two-stage screening protocol, where articles were first screened based on the title and abstract, then by full-text evaluation against inclusion criteria. Chosen studies were required to (i) discuss the type and changes of miRNA, (ii) the altered phenotype and (iii) both the positive and negative effects of the miRNA changes, if any. The initial search yielded 130 records. After further screening based on the inclusion criteria, 53 articles were retained for in-depth analysis.

## Biogenesis and mechanism of miRNA

Biogenesis of miRNA has canonical and non-canonical pathways [7]. In the canonical pathway, RNA polymerase II transcribes the miRNA genes into long primary transcripts called pri-miRNAs, which contain a hairpin structure [7]. Next, the hairpin is cleaved by a microprocessor complex which comprises Drosha and DiGeorge Syndrome Critical Region 8 (DGCR8) to produce precursor-miRNA (pre-miRNA). Exportin5/RanGTP exports the pre-miRNA to the cytoplasm and RNase III enzyme (Dicer-1) processes it to produce the mature miRNA duplex. Either a 3’ or 5’ strand of the duplex is loaded into the Argonaute family of proteins to form a miRNA-induced silencing complex (miRISC). For the non-canonical Dicer-independent pathway [1], the small hairpin RNA (shRNA) is initially cleaved by Drosha to generate short RNAs. They are then exported to the cytoplasm via Exportin5/RanGTP. Afterwards, the shRNA is matured by Argonaute 2 protein without the help of Dicer, which is a molecular scissor. This produces a mature miRNA that is ready to form the miRISC complex to regulate gene expression. A special class of miRNAs, which originate from introns (non-coding pieces of mRNA) that are spliced, is exported by Exportin to the cytoplasm and matured by Dicer, without the help of Drosha. Both canonical and non-canonical pathways produce the miRNA-induced silencing complexes, which involve the miRNA, the Argonaute and GW182 proteins. The complexes are ready to have a silencing effect on genes, such as translation repression.

In the miRNA-mediated gene silencing, miRISC usually binds to complementary sites in the 3’ untranslated region (UTR) of the mRNA [8]. Within the 3’ UTR, sites located near the stop codon or closer to the poly-A tail tend to have a more potent effect on silencing. MiRISC’s potency also increases when the binding sites are located near A/U rich regions. Cooperative interactions between miRNA-binding sites in 3’ UTRs were found to be crucial for the effects of miRNA-mediated silencing, suggesting that miRNA-binding sites that are close to each other often led to a more potent effect. In 3’ UTRs, it was also filled with RNA-binding proteins (RBPs). Both cooperation and competition effects arose between their interactions, such as loosening RNA structures or RBPs preventing miRISC from attaching. Together, these interactions determine whether an mRNA gets translated into protein or degraded.

According to Jungers & Djuranovic (2022), miRISC uses base-pairing to induce post-transcriptional effects [9]. It utilizes an imperfect base pair complementarity to bind to specific sequences, which were found mostly in 3’untranslated regions of target mRNAs, leading to repression of the gene expression of the transcript. MiRISC exerts its silencing function mostly through translational repression and mRNA decay. Its mechanism involves inhibition of translation, followed by deadenylation, decapping and decay of mRNA transcript [10]. MiRNAs impair the association of mRNAs to 40S or 80S ribosomes, where translation of protein occurs. Without enabling mRNAs to reach the production site, peptide bond formation is inhibited, which suppresses protein synthesis. MRNA possesses a long poly-A tail which protects the mRNA from nuclear decay and facilitates its maturation before exporting to the cytoplasm. With the carboxyl terminal domain of GW182 of miRISC, also referred as silencing domain, effector proteins are recruited, including deadenylases and mRNA decapping factors [9]. The cytoplasmic polyA binding protein (PABP), which interacts with GW182, promotes deadenylation through recruitment of the CCR4-NOT and PAN2-PAN3 deadenylase complexes. It was also suggested that significant poly-A tail shortening triggers transcript decapping by DCP1/2. When the polyA tail is shortened to 20 adenosines out of the original 60 adenosines, the mRNA would be prone to being decapped. Following the cap removal, the transcript eventually becomes degraded by exosome or exoribonuclease 1 (XRN1).

## miRNA mechanism underlying reproductive impacts

### miRNA direct impact on reproduction

The impact of the miRNA changes is not strictly limited to future generations, but the present generation as well. Since miRNAs are non-coding RNAs that suppress target gene expression post-transcriptionally, they can have a huge impact on reproductive processes or the structural formation of the gonad. Tahmasbpour Marzouni et al (2022) mentioned that changes to the miR-34 family, specifically miR-34a-c, can cause impaired spermatogenesis; as this miRNA is altered, sperm cell abnormalities might occur, lowering fertility [11]. Other than sperm abnormalities, miRNA changes can also cause hormone imbalance, which contributes to sperm production.

Exposure to bisphenol A (BPA) to rats have altered testicular miRNA expression, such as miR-361–5p and miR-203a-3p [12]. BPA also caused damage to the testicular structure, leading to a decrease in testosterone and luteinizing hormone (LH) levels. As LH stimulates testosterone synthesis in the Leydig cells, a reduction in the level of both hormones will affect spermatogenesis, possibly lowering the sperm count. miR-221 and miR-222 can induce differentiation of cells, loss of their function caused by retinoic acid exposure will lead to stem cell capacity loss [13]. Suppression of testosterone synthesis can also be due to the downregulation of steroidogenic enzymes such as CYP11A1 and StAR, lowering sperm motility and causing abnormal morphology [14]. Changes in the miRNA profile will affect reproduction in different ways, and these effects may persist and cause transgenerational effects in future generations, as outlined in the following section.

### Effect on gonadal development

Gonadal development is a process in which reproductive organs are produced through various processes, including the indifferent stage, genetic switch, and differentiation between males and females. During the indifferent stage, primordial germ cells (PGCs) are involved, they will migrate towards the primitive sex cords and further contribute to gonadal development. Changes in PGC differentiation will affect the animal’s growth, including the reproductive organs. In humans, Hwang et al (2026) pointed out that females with endometriosis had an altered miRNA expression profile including miR-21, miR-146a, and the miR-200 family, which was associated with elevated serum BPA levels [15]. These miRNAs are involved in processes such as granulosa-cell survival, inflammatory signaling, follicular remodeling, and steroidogenesis. So, the alteration of these miRNAs may contribute to ovarian dysfunction, possibly relevant to endometriosis. Importantly, miR-21 and miR-146 families are the shared miRNA regulators in studies of vinclozolin, BPA, and cadmium. Although the outcomes differ, this might represent the common molecular responders to environmental toxicants (Figure 1). Notably, miR-21 suppresses PTEN, a phosphatase that attenuates phosphatidylinositol 3-kinase (PI3K) signaling by dephosphorylating PIP3 and thereby limiting AKT activation [16]. Loss of PTEN-mediated inhibition enhances cell growth and survival. When tissue such as the uterine lining proliferates ectopically, this dysregulated growth can further compromise fertility and alter gonadal structure. Upregulation of certain apoptosis-related miRNAs can cause ovarian granulosa cell apoptosis under cadmium exposure. Sun et al (2023) discovered that after exposing P0 with cadmium, both the F1 and F2 generations had a differential miRNA expression pattern such as miR-92a-2–5p [17]. This miRNA is responsible for apoptosis regulation via the downregulation of anti-apoptosis gene, Bcl-2. In addition, Sun’s group found that miR-92a-2–5p was highly expressed in the F2 and F3 generations when compared to the F1 generation, suggesting that its high expression might be related to granulose cell apoptosis, which can be further transmitted to future generations.

**Figure 1:**
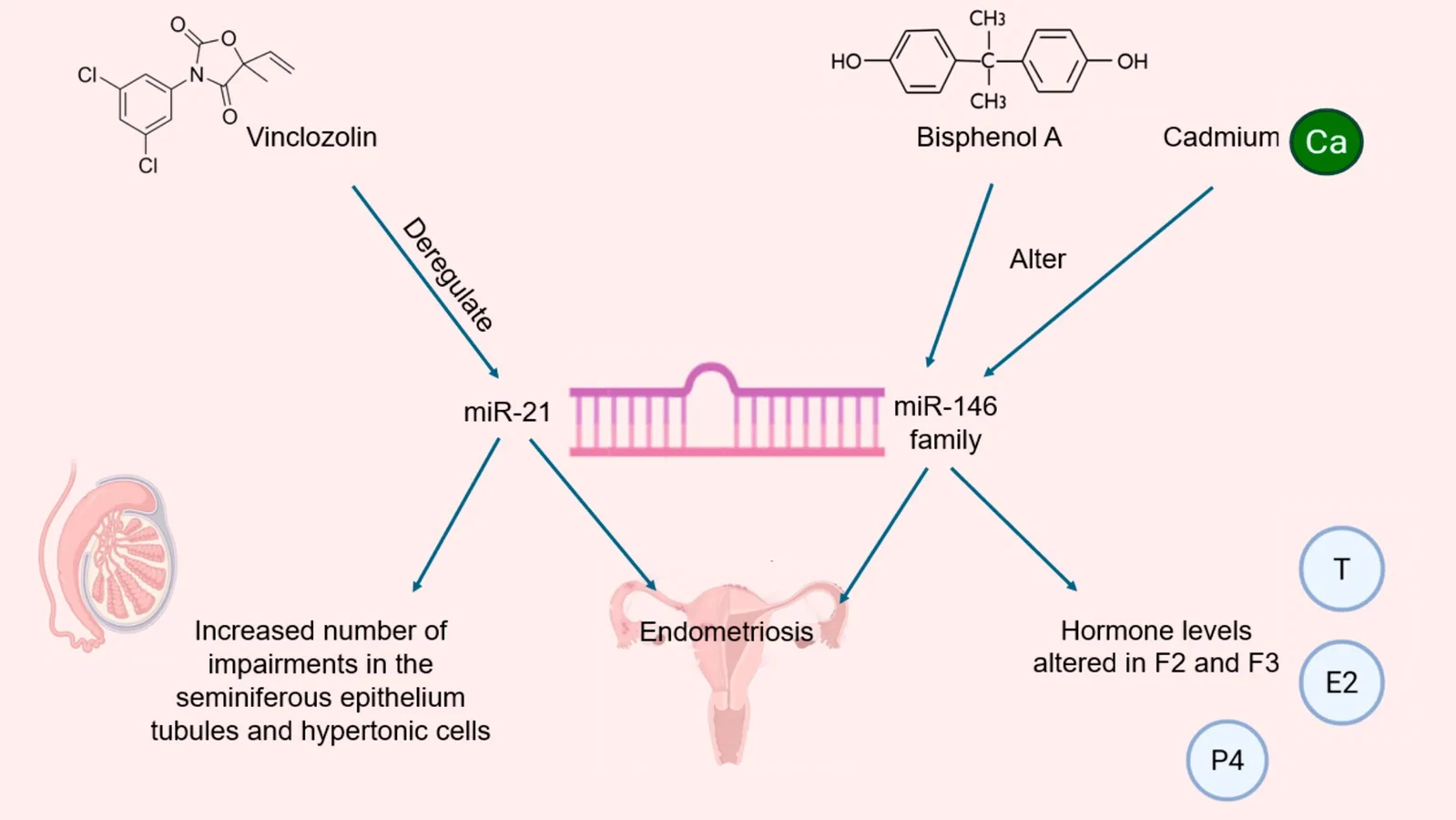
Possible roles of miR-21 and miR-146 families on inducing endometriosis caused by different environmental toxicants. They can also cause other health problems, including impairments in the seminiferous epithelium tubules, or alteration of the hormone levels in F2 and F3 generation [15,17,18].

The miRNA effect on mice gonadal development was investigated. Parental exposure to vinclozolin, an endocrine disruptor, caused an increased number of impairments in the seminiferous epithelium tubules and hypertonic cells, this was observed in all three generations of male mice [18]. The same article showed that this exposure caused a reduction in the number of PGCs per testis, from 57% in F1 to 61% in F2, the same trend can be applied to the low-dosage group. Vinclozolin exposure caused deregulation of miR-23b and miR-21, which was suggested to be associated with the observed gonadal impairment. Let-7 was also deregulated, which is involved in regulating BLIMP1, a transcription factor that contributes to the specification of PGCs [18]. A similar mice study by Liu et al (2022) that dexamethasone exposure reduces the expression of steroidogenic acute regulatory (StAR) protein and the 3β-HSD gene, resulting in an enlarged intra-testicular interstitial region and shrunken nuclei [19]. These effects persisted into the F3 generation. The transgenerational inheritance was mediated by PDE, which downregulates miR-466b-3p, leading to increased HDAC7 expression. In the absence of miR-466b-3p–mediated suppression, HDAC7 is upregulated, thereby reducing StAR protein levels as described above. This changes the epigenetic marks in the germ cells, allowing them to escape the reset process during germ cell maturation and early embryo development. Gonadal development disruption can also be observed in fish. Hypoxia exposure can lead to reproductive impairment in female marine medaka [20]. The experiment found that when comparing the F0 to F2 generation, four miRNAs were dysregulated, and three miRNA-mRNA pairs (novel miRNA-525-DIAPH2, novel miRNA-525-MYOCD, and novel miRNA-525-RAI14) were identified, which could play an epigenetic role in hypoxia-induced reproductive impairment. For example, deregulation of miR-525 alters DIAPH2, a key regulator of actin cytoskeleton organization and ovarian cell function, thereby contributing to ovarian dysregulation.

### Effect on steroidogenesis and hormone changes

Since miRNA is demonstrated to cause changes in gonadal development and steroidogenesis, the hormones produced may also be affected. Luo et al (2023) performed an experiment on rats, aiming to find out the effect of cadmium exposure on hormone synthesis [21]. Their results showed that the F2 females had decreased serum estradiol and progesterone levels, whereas the F3 females had increased levels, involving miR-124–3p, miR-133a-5p, miR-150–5p, let-7a-5p, miR-134–5p, and miR-30c-1–3p. In a rat study on the transgenerational effects of cadmium exposure, Sun et al. (2023) reported altered estradiol and progesterone levels in both the F2 and F3 generations [17]. The miRNAs implicated included miR-27a-3p, miR-27b-3p, miR-146 family, and miR-10b-5p. Dose-dependent hormonal changes were observed in both F2 and F3 generations. Notably, both experiments identified the same hormone-synthesis genes including StAR, Cyp11a1, Cyp17a1, Cyp19a1, and Sf-1, despite differences in the specific miRNAs involved. Dysregulation of these genes disrupts cAMP/PKA signaling, thereby altering estradiol and progesterone synthesis across generations (Figure 2). A study by Liu et al (2022) also pointed out that gonadal development changes could lower StAR protein, 3β-HSD gene expression, and testosterone levels, further strengthening the theory that changes in gonadal development can affect hormones in the body [19].

**Figure 2:**
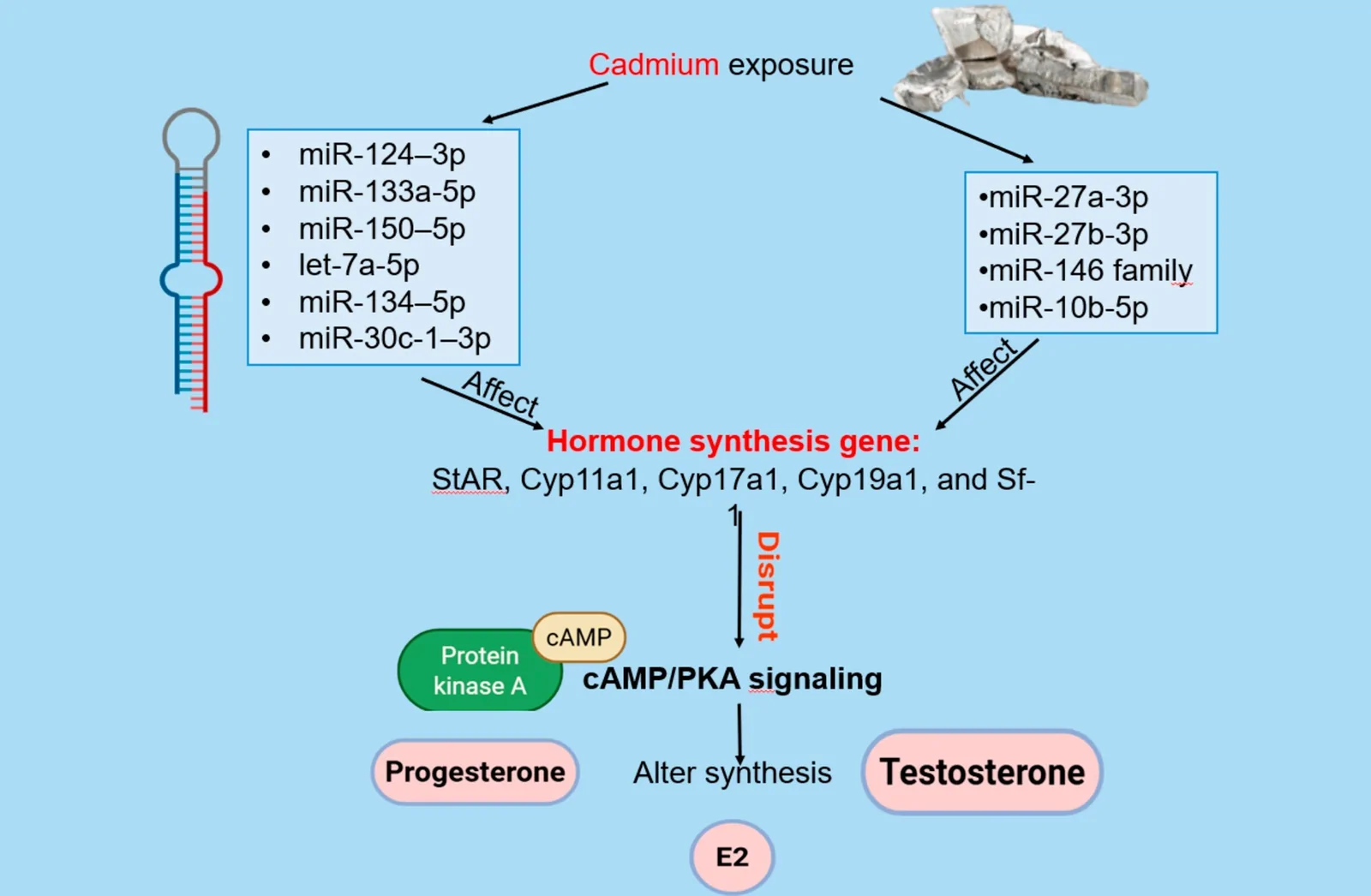
Cadmium exposure can cause alternation of two groups of miRNAs, eventually disrupt the cAMP/PKA signaling, leading to alternation of hormone synthesis [17].

### Effect on spermatogenesis and sperm parameters

Coordinated interactions between germ cells, Sertoli cells, Leydig cells, and the hormonal environment are required for normal spermatogenesis, miRNA changes in any of these may affect sperm production and quality, such as sperm morphology. Impaired seminiferous epithelium was observed when rodents were exposed to micro- and nano-plastics, causing downregulation of spermatogenic markers and also germ cell loss [22]. Using chicken as a model, Gong et al (2021) investigated the regulation of transforming growth factor beta 2 (TGFB2) by miR-301a-5p during spermatogenesis [23]. Results showed that miR-301a-5p may inhibit the expression of TGFB2, thereby affecting the TGFB pathway. Since TGFB2 is highly expressed in Sertoli cells and contributes to the maintenance of adult spermatogenesis, disruption of the TGFB pathway can affect spermatogenesis in humans. This will further influence sperm quality.

In a Zebrafish study performed by Fu et al (2024), high expression of NC_007115.7.7_998 413 was observed to inhibit sperm flagella formation, affecting the expression of spermatogenesis associated 2 (spata2), leading to sperm malformation and reduced male fertility [24]. The authors suggested that this miRNA may have potential as a biomarker for spermatogenic disorders. A mice study performed by Yokota et al (2025) investigate the impact of silver nanoparticles (AgNP) on the spermatogenesis [25]. They discovered that the male mice from both high- and low-dose exposure groups produced a high proportion of abnormal sperm, such as abnormalities in the head and tail in the F2 generation. The percentage of hyperactive sperm was also lower when compared to the control group. When the mice were exposed to AgNP, the testicular miR-465c-1 was upregulated and miR-106a-5p was downregulated. These changes were associated with disrupted germ-cell development, hence affecting sperm quality of the present and future generations. As obesity affects the body’s health, it can also affect sperm quality. Zhang et al (2024) conducted an experiment with obese patients who suffered from impaired reproductive capacity with potential transgenerational consequences [26]. They pointed out that obesity could cause an increase in sperm miR-135b; this miRNA was predicted to regulate genes involved in impairing iron absorption, affecting fertilization, sperm development, and viability. However, further investigation is needed to confirm the direct correlation between miR-13b and these reproductive outcomes.

### Effect on oogenesis, oocyte parameters and fertility

Similar to spermatogenesis, oogenesis is the process of oocyte production in the ovaries. But since both of them are considered gametogenesis, the miRNA alteration will affect both sperm and oocytes. A study in female mice investigating the long-term reproductive toxicity of dexamethasone reported a reduction in the number of primordial follicles and an upregulation of miR-17–5p in F1 offspring [23] Across the F1 to F3 generations, Forkhead Box L2 (FOXL2) and cyclin-dependent kinase inhibitor 1B (CDKN1B) were consistently downregulated, accompanied by decreased binding of FOXL2 to the CDKN1B promoter region. Furthermore, the activity of dexamethasone was shown to involve glucocorticoid receptors (GR), leading to diminished ovarian reserve in offspring. This effect could contribute to transgenerational inheritance, as increased miR-17–5p expression was observed in oocytes. Oogenesis can also be disrupted by endocrine-disrupting chemicals (EDCs), such as bisphenol A (BPA). According to Hwang et al (2026), BPA can cause overexpression of miR-21 in mice, which can impair ovarian granulosa cell function; there was an abnormal level of the miR-200 family, which was associated with polycystic ovary syndrome (PCOS) pathogenesis, a condition that causes hormone imbalances and metabolic disorders [15]. They are also involved in processes of follicular remodeling and oocyte maturation. Furthermore, they pointed out that parental exposure to BPA reduced ovarian follicle number in F1 to F3 female mice, with their fertility reduced [15]. In addition, mice exposed to vinclozolin had alteration of miRNA such as miR-23b, causing an increase in germ cell apoptosis, and reduced ovarian reserves. [18]. These outcomes will lead to premature ovarian insufficiency that may decrease fertility.

### Transgenerational inheritance

Although current evidence for transgenerational miRNA effects on the reproductive system derives from studies involving different types of substances, these findings collectively suggest that miRNA profile alterations in a species can contribute to the transmission of reproductive disorders to future generations. Importantly, in these models the offspring were not directly exposed to the substances, confirming that their exposure was not a confounding variable. As Brieño-Enríquez et al (2015) pointed out, alterations in PGC differentiation can be observed in the F3 generation, which can further affect sperm miRNA profiles, affecting PGC function, such as their growth and reproductive organs [18]. In gonadal development, cadmium exposure of P0 mice affects the regulation of apoptosis in the F3 generation due to Bcl-2 being downregulated, causing granulosa cell apoptosis [17]. In addition, overexpression of miRNAs might impair ovarian granulosa cell function in mice, reduce their ovarian follicle number, and eventually impact fertility [15].

## miRNA mechanism underlying obesity

### Correlation between miRNA activities and obesity

Obesity is caused by adipocyte hypertrophy and hyperplasia, meaning enlarged and increased adipocytes. Cirillo et al. (2019) found that miRNA dysregulation correlates with altered adipogenesis and insulin resistance, leading to obesity. Inhibition of miR-15a enhanced hyperplasia while miR-210 stimulated the formation of hypertrophic adipocytes [27]. MiR-448 was also involved in the expression of adipogenic genes and triglyceride accumulation [27]. Cirillo et al. (2019) reported that various miRNAs were upregulated, such as miR-142–3p and miR-122, while others were downregulated, such as miR-221 and miR-28–3p, resulting in increased body weight and insulin resistance with reduced proinflammatory cytokines [27].

Apart from adipogenesis, Zhao et al. (2026) revealed that miRNA/Glucagon-like peptide-1 (GLP-1) played a critical role in glucose homeostasis, appetite regulation, and central nervous system functions [28]. GLP-1 was involved in stimulating insulin secretion, appetite regulation, enhanced lipid oxidation, and increased energy expenditure [28]. MiRNAs could either attenuate GLP-1R expression or mediate GLP-1R transcriptional adaptations. With reduced GLP-1R expression, incretin signaling was weakened to reduce production of insulin, increasing obesity risk; increased expression of GLP-1R could promote metabolic signaling and insulin sensitivity [28]. For instance, miR-7 targeted β-Arrestin 1 (βARR1) and reduced its expression, lowering cAMP production by suppressing the GLP-1 signaling. This led to reduced GLP-1 stimulated insulin secretion. miR-27a targeted AMPK ameliorated diabetic lung fibrosis [29]. This demonstrated how epigenetic regulations create a dynamic balance in metabolic signaling and functions.

According to Sridhar et al. (2026), MiR-155 is an exosomal miRNA secreted by enlarged adipocytes of obese mice [30]. It impairs insulin signaling in peripheral tissues by targeting PPAR and KLF4 [30]. Elevated circulating miR-155 levels were observed in obese mice with diabetes, which was transferred by adipocyte-derived macrophages to bone marrow derived macrophages [30]. Pro-inflammatory cytokines such as IL-6 were also increased. Besides, miR-155 was shown to encourage the polarization of pro-inflammatory M1 macrophages, which affects adipocytes in a paracrine manner, inhibiting their insulin sensitivity and uptake of glucose [30]. In addition to miR-155, miR-92a was also upregulated in obese mice, which acts on the gene KLF2 and downregulates miR-483 level of expression [31]. Loss of miR-483 removes repression of its target genes, resulting in metabolic dysfunction, and that includes alteration in the metabolism in blood lipids and blood glucose. These findings indicate that miRNAs play a role in affecting metabolic functions under the influence of obesity and diabetes.

### Effect of miR-148a

MiR-148a plays a key role in adipogenesis regulation and acts as a critical epigenetic regulator in the pathogenesis of obesity. Acting as a post-transcription silencer, MiR-148a works by repressing the gene Wnt1, and that typically accelerates the lipid accumulation and adipocyte differentiation process [32]. It is also agonistic with PPAR-dependent transcription, which is a key driver of fat cell formation [33]. Level of miR-148a typically increased during the differentiation of human adipose-derived mesenchymal stem cells. Dysregulation of miR-148a could lead to excessive adipose storage and accumulation, which results in obesity. A high fat diet would increase the expression of miR-148a in adipose tissue of obese people and mice. Engin & Engin (2024) found that miR-148a also functions in intercellular communication [34]. When fat cells are stressed or enlarged, adipocyte-derived exosomes are released, and miR-148a goes inside the exosomes. They act as a messenger that exerts an effect on other cells. Consequently, altered miRNA expression during the perinatal period will increase the risk of transgenerational inheritance of obesity (Figure 3). In a study by Li et al. (2020), exposure to pollutant PDE also induced hypercholesterolemia in adult male mice offspring [35]. It was found that miR-148a expression could be extended from intrauterine to adulthood of F3 male mice, and increased degradation of mRNA LDLR were found in F3, resulting in hypercholesterolemia.

**Figure 3:**
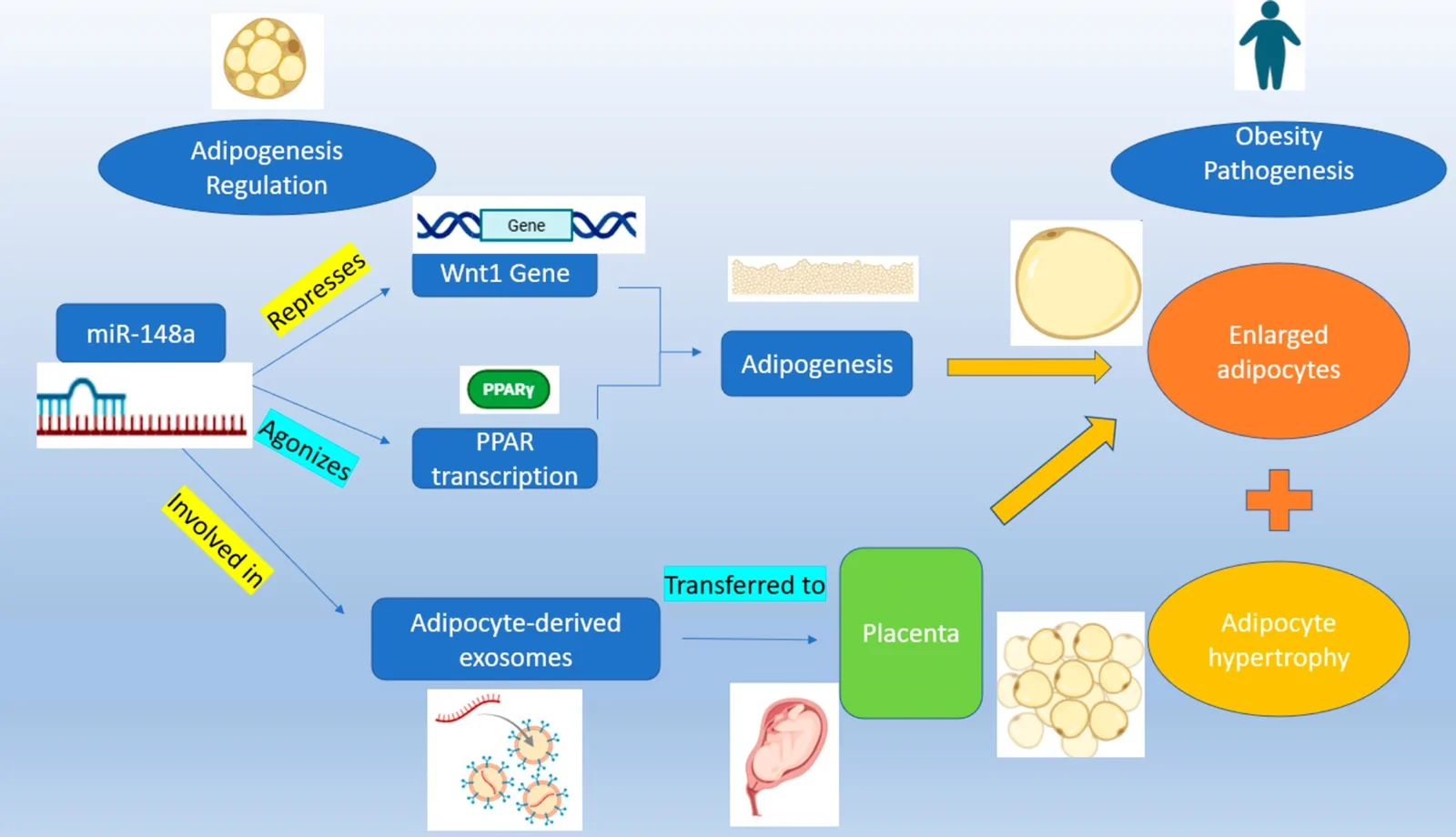
miR-148a played a key role in adipogenesis regulation through repressing Wnt1 gene and agonizing PPAR transcription. miR-148a was involved in adipocyte-derived exosomes, which were transferred to the placenta. With enhanced adipogenesis, adipocytes enlarged and increased in number, contributing to obesity [32,33 ].

### Effect of miR-223 upon exposure to obesogen

Exposure of pregnant F0 mouse to tributyltin (TBT) causes male descendants to be more prone to obesity and diverts mesenchymal stem cells (MSCs) toward the adipocytic lineage [36]. TBT has been found to be an environmental obesogen. Chang et al. (2023) conducted an experiment by exposing pregnant mouse (F0) MSCs to TBT, and then their transcriptomic profile was determined [36]. MiR-223 was shown to increase in MSCs upon exposure, and it reduced the expression of several target genes, ZEB1, NFIB, and FOXP1, which normally inhibit adipogenesis [36]. PPAR γ was found in adipose tissue and regulates adipogenesis. Exposure to TBT caused PPAR γ to form a complex with RXR and bind to the miR-223 promoter, activating the transcription of miR-223 [36]. Elevated miR-223 would persist across generations, particularly in F2 and F3 of mice exposed to 50nM of TBT, leading to transgenerational obesity by promoting adipogenesis and fat cell accumulation [36].

### Effects of miR-19b and miR-29a upon paternal high fat diet

Fullston et al. (2013) investigated the effect of diet-induced paternal obesity on metabolic health of mice offspring [37]. It was found that paternal obesity could deteriorate the metabolic health of the two resultant generations via molecular changes in testes and sperms. Paternal high fat diet (HFD) dysregulates the miRNA content of spermatozoa [38]. Across the study from Fullston et al. (2013), which targeted on mice, the F1 offspring had increased body weight and higher glycemia in response to glucose and insulin, while obesity was specifically developed in females inherited from male mice (F0) with high fat diet [37]. Among the F2 offspring, females showed insulin resistance while males were found to have glucose intolerance and obesity [38]. The testes from HFD and control-diet F0 fathers were analyzed for miRNA content. It was found that 23 miRNAs were deregulated due to HFD, with 4 miRNAs particularly affected in spermatozoa. These deregulated sperm miRNAs were predicted to target 50 mRNAs, which were shown to have enrichment in lipid metabolism and spermatogenesis [38]. With miRNAs alteration inherited to F2 that was unexposed to high fat diet, the altered metabolic phenotype is regarded as a transgenerational effect. Another study from Grandjean et al. (2016) showed that both male and female F1 offspring from HFD F0 fathers had increased body weight [39]. Moreover, male F1 offspring showed altered fasting blood glucose and higher glycemia in response to both glucose and insulin, which were regarded as signs of insulin resistance [39]. Next generation sequencing analysis confirmed the deregulation of 7 miRNAs in male gonads of HFD fed mice, with miRNA-19b and miRNA-29a as the two most abundantly deregulated in the testes and spermatozoa [39]. The experiment of microinjection of MiR-19b into fertilized mouse embryo (F1) was found to increase fasting glucose levels and might impair the glucose tolerance and insulin sensitivity of the F2 offsprings [39] (Figure 4) Furthermore, although the F2 generation was fed with a normal diet, changes in metabolic phenotypes take place due to the transmission of altered miRNAs from F1. This study indicated that F2 offspring reproduced by F1 offspring with microinjection of miR19 displayed increased body weight, altered blood glucose level and insulin resistance through epigenetics inheritance [39].

**Figure 4:**
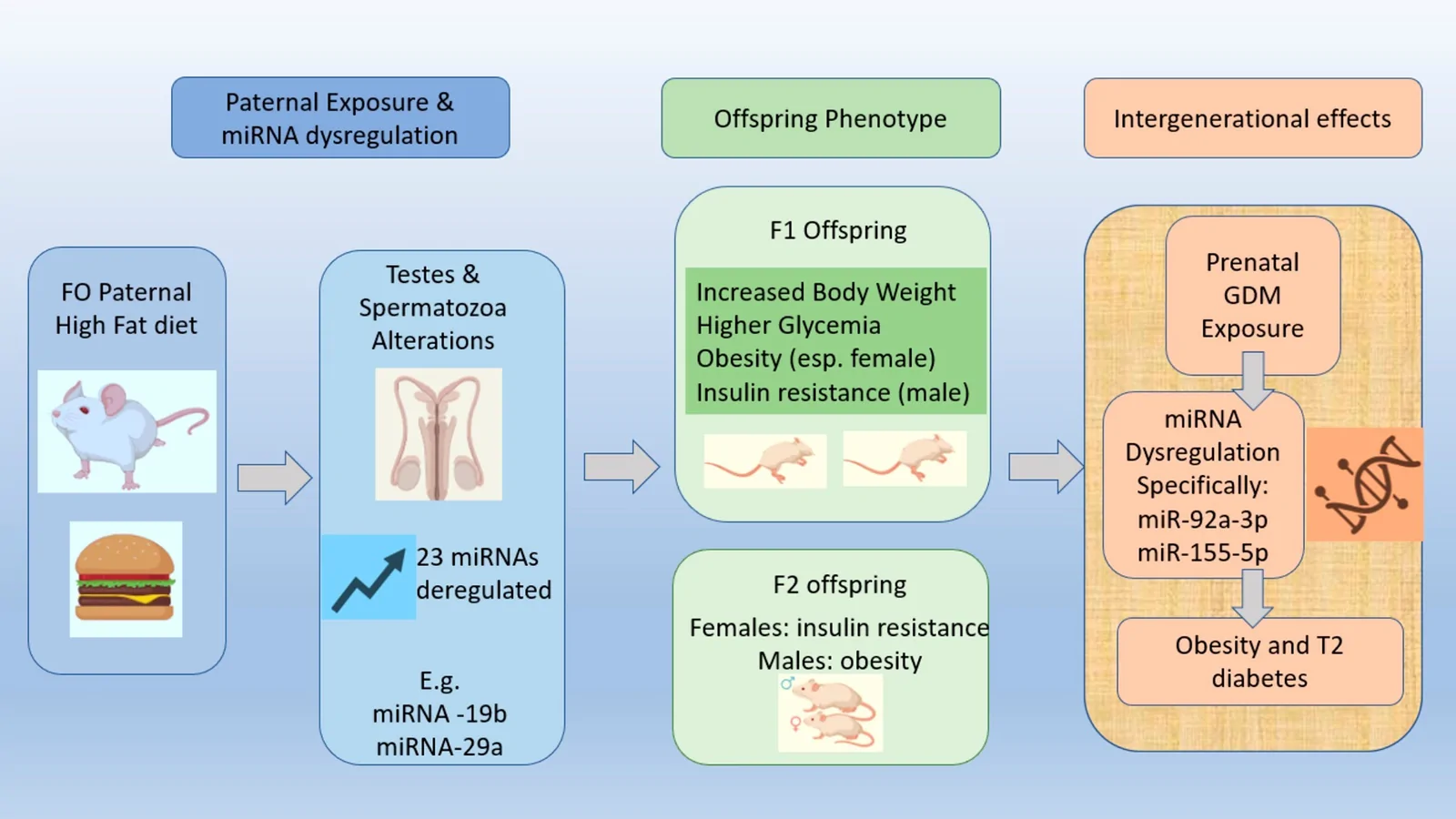
Paternal high fat diet could dysregulate miRNA, causing the inheritance of obesity, hyperglycemia and insulin resistance trans-generationally [37,38]. Prenatal GDM exposure would cause intergenerational inheritance of obesity and type 2 diabetes [43].

MiR-19b plays a key role in the progress of mediating inflammation in adipose tissue. It positively regulated the expression of inflammatory cytokines, such as interleukin 6 (IL-6) and 1 β (IL- β) [40]. The excess of macronutrients in adipose tissues stimulates them to release inflammatory mediators. With a rise in miR-19b during obesity or hyperglycemia, it binds to SIRT1 protein’s mRNA, lowering SIRT1 levels [40]. SIRT1’s knockdown promotes the pro-inflammatory effects of cytokines [40], resulting in chronic inflammation in adipose tissue. Persistent adipose inflammation would deteriorate insulin resistance, leading to the progression of obesity-related metabolic complications [41]. For miR-29a, high fat diet leads to a reduction in levels of miR-29a and elevated expression of glucogenic enzyme glucose-6-phosphatase 1 (G6pc1)b [42]. Knockdown of miR-29a in mice increased G6pc1 expression, which serves to induce glucose intolerance and insulin resistance [42]. Sperm miR-29a were consistently reduced in high glucose or high fat F0 and F1/F2 group [42], suggesting the transgenerational epigenetic transmission of obesity or hyperglycemia through miR-29a and G6pc1 interaction.

### Intergenerational effects of gestational diabetes on obesity

Hromadnikova et al. (2020) supported the hypothesis that disease-associated miRNAs would be altered in children descending from pregnancies affected by gestational diabetes [43]. Alterations in gene expression of miRNA associated with diabetes were discovered in whole peripheral blood of children aged 3–11 descending from gestational diabetes (GDM) complicated pregnancies. Almost all miRNAs with a few exceptions were upregulated, which differed from children of normal parental lineage [43]. The miRNA profile differed in expression and was correlated with obesity, hypertension or valve problems and heart defects in the descendants [43]. The miRNA expression patterns in placental and germ cells have been implicated in fetal programming, and increasing evidence is considering the function of miRNAs in mediating intergenerational epigenetic inheritance.

The effects of fetal programming that predisposed the affected children to later development of diabetes were examined. Out of 29 disease-associated mRNAs, 23 were found to be deregulated in children exposed to prenatal GDM [43]. Almost all miRNAs with some exceptions were upregulated in whole peripheral blood of affected descendants. 88.14% of children (F1) were found to have aberrant profiles [43]. For example, miR-92a-3p and miR-155–5p deregulation were observed in children with a prenatal exposure to GDM [43], confirming the hypothesis that affected children have an altered miRNA expression profile associated with prenatal GDM. To conclude, the offspring with prior GDM exposure would have an increased risk of obesity and other metabolic disorders, such as diabetes. (Figure 4) The current studies only provided evidence about the intergenerational effects of prenatal GDM on the F1 offspring. Maternal miRNAs contribute to altered metabolic functions as they are transferred from maternal tissues into circulation and across the placenta to the fetus. The fetus may have macrosomia which associates with a higher risk for subsequent childhood obesity and future metabolic disorders due to persisted fat cell accumulation and increased insulin resistance [44]. However, the mechanism of GDM transgenerational effects remains limited.

### Effect of miRNA on complications of obesity

Obese male mice were shown to have altered inflammation and iron regulation. According to Lopez et al. (2018), inflammation is regulated by miR-155 and miR-21 in immune cells, while miR-122 in plasma regulates iron metabolism [45]. MiR-155 gene is located on chromosome 21 and was demonstrated to promote macrophage polarization, which produces large amounts of pro-inflammatory cytokines [45]. MiR-155 silences genes that repress the production of proinflammatory cytokines [45]. Furthermore, miR-122 targeted at Human Hemochromatosis Protein (HFE) and Hemojuvelin suppressed them to reduce transcription of hepcidin (HAMP) [45]. Lower levels of HAMP led to deregulated iron and forced them to deposit abnormally [45]. As these variations in miRNA were altered in sperm of obese people, altered inflammation and iron deregulation would be passed to the next generation through paternal epigenetic inheritance.

## miRNA mechanism underlying mental health issue

### Correlation between stressed phenotype and miRNA activity

Khan et al. (2026) revealed that miRNAs played a key role in the neurotransmitter signaling pathways, which were critically disrupted in various psychiatric conditions [46]. For major depressive disorder, miRNAs affected the levels of serotonin and brain-derived neurotrophic factor (BDNF), both of which were pivotal in depression pathophysiology. According to Wei, Wu & Chen (2024), neurotrophic factors are a family of proteins that provide nutrients for neuronal growth and survival [47]. MiR-206 significantly impacted the expression of BDNF, causing dysregulation of neurogenesis and prevalence of mood-related behaviors [47]. Another mental health disease affected by miRNA was anxiety. Anxiety also arose from changes in BDNF levels, and key regulators included miR-15a and miR-17–92 [46]. Gene expression exerted by miRNAs included weakened synaptic density, low BDNF levels, and impaired neurotransmission, resulting in various mental health impairments [46]. Abdolahi et al. (2022) proposed the interplay between neurotrophins and miRNAs in neuropsychological disorders [48]. MiRNAs act as regulators of gene expression, ensuring that neurotrophin signaling is normal [48]. They sustain positive and negative feedback loops, maintaining healthy brain function [48]. If the levels of miRNAs concerned are dysregulated, synaptic plasticity would be impaired, causing mood dysregulations [48].

### Effect of paternal chronic stress on offspring’s mental health status

Chronic social instability during adolescence and early adulthood induces persistent behavioral alterations, including enhanced anxiety and social deficits. Saavedra-Rodriguez & Feig (2013) used a group of mice to evaluate long-term behavioral effects caused by stress exposure from postnatal day 27 to 76 [49]. The study showed that it was transmitted predominantly to females across at least three generations, with the F1 generation associated with elevated serum corticosterone level and other reasons in the F2 and F3 generations [49]. Presumably, epigenetic changes in the brains of F1 females occurred in utero in response to the stressed environment, which was attributed to a rise in corticosterone [49]. The F1 and F2 fathers transmitted the altered behaviors to their F2 and F3 female progeny. Instead of having elevated corticosterone, findings suggested that germline epigenetic changes had taken place in males that passed on the altered traits to their female offspring [49].

The transgenerational inheritance of social instability deviated traits could be explained by adult males (F1) inheriting reduced sperm miR-34/449 levels, which were passed down to females (F2 and F3), resulting in elevated anxiety and impaired sociability [50]. Evidence also supported that restoring miR-34c levels in a preimplantation embryo (PIE) prevents elevated anxiety and defective sociability [49]. MiR-34/449 can inhibit the expression of several mRNAs, including CDK6, c-MYC, HDAC1, and BCL-2 [50]. Experiments also revealed that inducing miR-34c levels in PIE enhances the expression of its own gene and that of miR-449 in these cells [50]. The effect of miR-34c could be explained by these amplification dynamics even though sperm contribute only a tiny fraction of the embryo. When sperm miR-34c was suppressed, the amplification loop ended, and this could be attributed to the male’s early life trauma exposure [50]. This correlates paternal stress to offspring developmental risks. (Figure 5)

**Figure 5:**
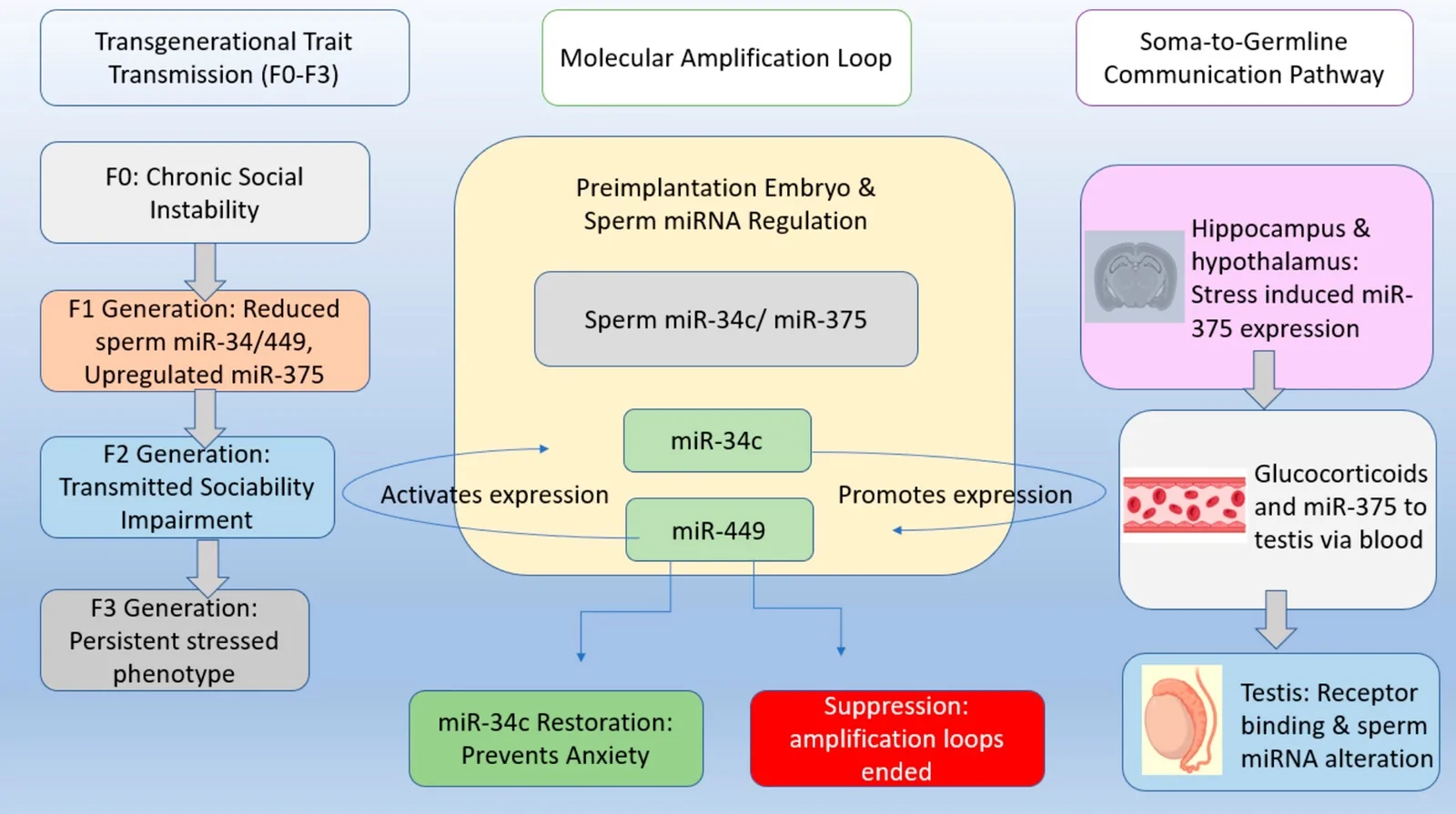
F0 chronic social instability could be transferred to F1-F3 through miRNA epigenetic regulations [49]. MiR-34c and miR375 were revealed to play a key role in trans-generation effects, altering the miRNA of sperms and passing the stressed phenotype [50].

Stress exposure in the F0 generation altered the sperm miRNA of F1, including miR-375, which is linked to stress regulation and metabolism. Findings of Sharma (2015) showed that several miRNAs, including miR-375, were upregulated in sperm of chronically stressed mice [51]. In a study on transgenerational inheritance of chronic social instability in mice, it was found that altered expression of miR-375 was also found in brain structures related with stress response, precisely the hippocampus and hypothalamus [51]. Notably, injection of sperm RNA isolated from stressed mice into fertilized mouse oocytes from unaffected females resulted in offspring with altered behavioral, metabolic, and molecular phenotypes [51].

It is also interesting to study the relationship between soma and germline communication, which could explain transgenerational inheritance. Newer findings did suggest that extracellular miRNAs in mammals possibly participate in soma to germline transfer, therefore mediating the inheritance of acquired characteristics. Studies showed that stress hormone glucocorticoids reach the testis through circulating blood, then bind to the receptors expressed in sperm and induce changes in miRNA expression [51]. Hippocampal miR-375 also reached the testes through circulation and triggered altered miRNA expression in sperm [51]. The relationship between stress hormones, brain-derived miRNAs, and exosomal transfer converges to form a plausible pathway for soma-to-germline information transfer [51]. (Figure 5) DNA methylation was observed in rodents, which correlated with altered emotions. Due to miRNAs’ potential effects as methylation regulators, hypermethylation in Mecp2 and hypomethylation in Crhr2 occurred [51]. The methylation marks were present in F1 sperm and persisted in F2 sperm, with hormones alteration and altered gene expression level in the stress-regulating brain region [51]. Both F1 and F2 offspring exhibited impaired long-term memory and amplified stress hormone signaling in adulthood [51].

### Diseases associated with stress induced by miRNA

King et al. (2025) suggested prenatal maternal stress (PNMS) as a major driver of adverse pregnancy outcomes and a risk factor for chronic illness in adulthood [52]. Exposure to PNMS can promote adult-onset disease by influencing fetal development through epigenetic modifications, particularly altered miRNA [52]. miRNA could regulate gene transcription activities and persist into adulthood [52]. Both DNA methylation and miRNA expression could affect brain development and increase psychiatric disease risk in offspring, suggesting that the placenta could play an important role in their etiology [52]. If PNMS happened during the critical time of primordial germ cell development, aberrant epigenomic patterns could be reprogrammed into the germline, propagating across several generations even in the absence of prenatal stress exposure in subsequent generations. If the developing embryo experiences direct exposure to the placenta (F1), germline development will become affected, which will later contribute to the F2 generation [52]. Due to epigenetic changes, preterm birth and adverse newborn outcomes might result. Ancestral stress also promotes behavioral changes, including anxiety-like behavior, depression-like phenotypes, and motor hyperactivity [52]. In addition, it was observed that F3 offspring had a reduced weight on postnatal day 1 and delayed sensorimotor development [52]. The compounding consequences of PNMS accumulate negative effects across generations.

### Therapies for miRNA-induced stress related diseases

Prenatal stress in rats could generate an epigenetic footprint. It caused a downregulation of the glucocorticoid receptor, which is used for stress mitigation with negative feedback [53]. While ancestral stress could influence the filial generations, it is crucial to identify therapies that could overcome transgenerational programming. Environmental enrichment (EE) during adolescence mitigated endocrine and neuronal markers of stress and improved miRNA expression, which was linked to stress regulation [53].

Environmental enrichment, which was targeted at mice in the study by McCreary et al. (2016), referred to adding scattering food treats and running wheels to stimulate a positive emotional state [53]. Deep sequencing revealed that 29 miRNAs could be differentially expressed in response to EE [53]. It worked by upregulating or downregulating their expression. McCreary et al. (2016) revealed that animals housed in the enriched environment demonstrated fold changes of the miRNAs, such as miR-182, which regulates neurotrophins signaling and axon guidance [53]. MiR-182 was found to be associated with depression-like behaviors and decreased hippocampal BDNF protein expression. McCreary et al. (2016) continued to explain that EE exposure could largely mitigate the stress-sensitive phenotype by modifying the hypothalamic-pituitary-adrenal (HPA) axis [53]. Reduced HPA axis activity may directly improve anxiety-like behavior [53]. Apart from reducing anxiety, EE could benefit stressed rats with improved skilled movement, learning and memory, and affective behaviors [53]. The present study concluded that EE is capable of improving stress response regulation and affective state in male offspring.

## Limitations

Despite the insights provided by this review, several limitations should be acknowledged. First, the literature search was conducted using PubMed alone, which may have resulted in the omission of relevant studies indexed in other databases. Second, the review was restricted to miRNA-related mechanisms; therefore, the potential contributions of other epigenetic mechanisms, such as DNA methylation and histone modifications, were not evaluated. Third, the review focused on only three categories of transgenerational effects, which may have limited its ability to capture the full range of outcomes associated with miRNA alterations. Fourth, the included studies used different animal models and environmental toxicants. This methodological heterogeneity makes direct comparisons between studies difficult and may limit the generalizability of the findings. Finally, differences in exposure dose, exposure duration, tissue examined, miRNA measurement methods, and outcome definitions prevented quantitative synthesis and limited the ability to determine consistent dose–response relationships.

## Conclusion

Collectively, the studies reviewed here suggest that alterations in miRNA profiles, whether induced by environmental exposure, high-fat diets, or parental stress, can be inherited across generations (Table 1). These changes can affect multiple systems in the human body. In reproduction, miRNA dysregulation can disrupt steroidogenesis, induce sperm and oocyte defects, and ultimately reduce fertility. Various factors, including gestational diabetes, high-fat diets, and obesity, may arise in one generation and be transmitted to the next. Prenatal stress can also be passed down to offspring, leading to conditions such as depression-like phenotypes and motor hyperactivity [52]. Although the specific transgenerational effects vary depending on the type of stimulus, a common theme emerges that miRNAs can modulate gene networks, contribute to apoptosis [17], germ cell differentiation [18], and more. Figure 6 summarizes the miRNA-mediated transgenerational effects discussed in this article. While further research is needed to clarify the precise relationship between miRNAs and transgenerational inheritance, advancing technologies are likely to increase the relevance of miRNAs as biomarkers across different domains, with potential benefits for multiple species.

**Figure 6:**
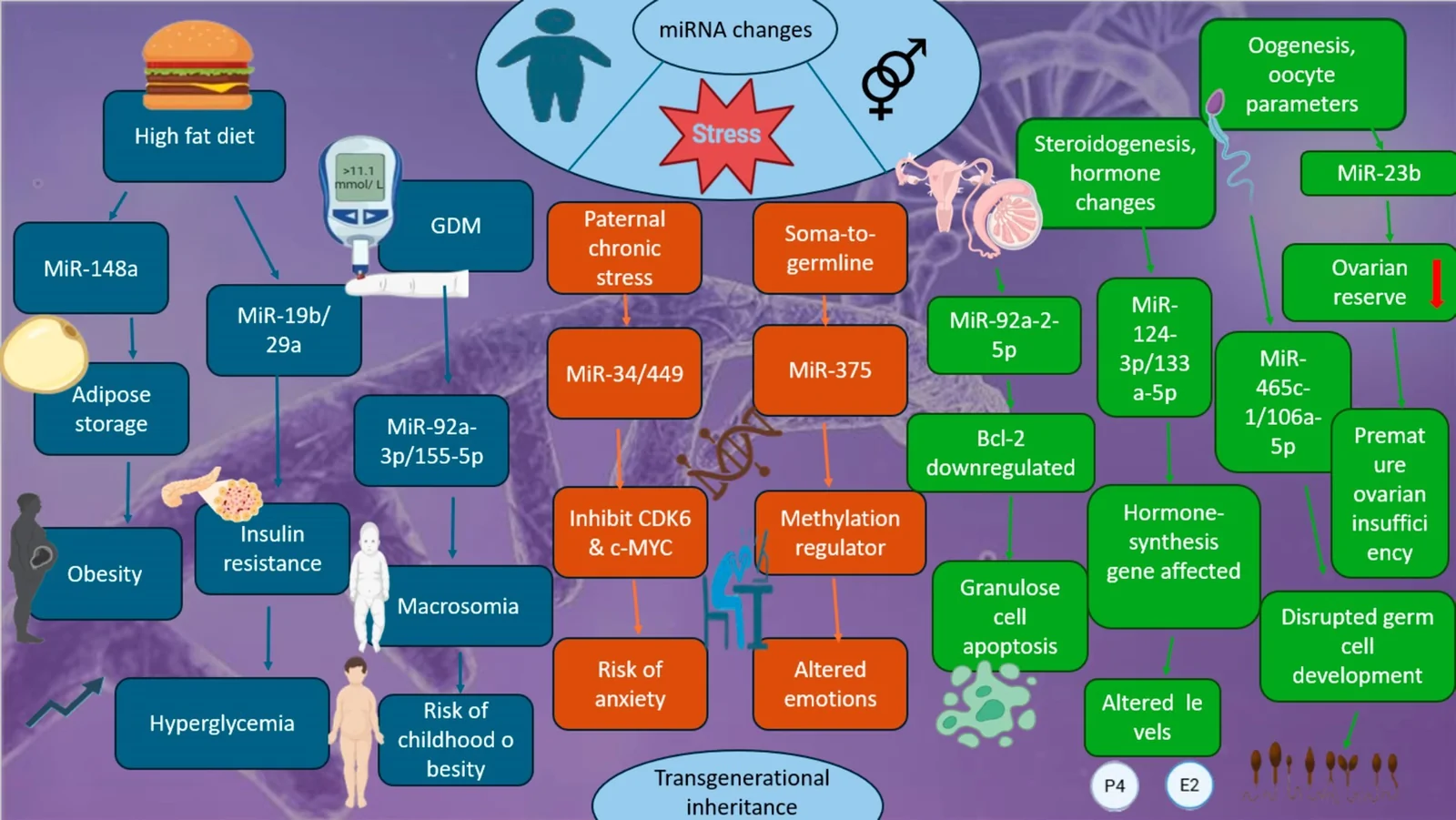
Summary of the key aspects discussed in this paper, including diabetes, stress, and reproduction. Selected miRNAs are listed to illustrate how alterations in their expression levels can lead to transgenerational effects [17, 18, 32, 33, 37, 38, 43, 44, 49, 50].

**Table 1:** Summary of transgenerational effects of miRNA.

Model	Environmental toxicant	Affected miRNA	Effect of miRNA changes	Affected protein/genes	The transgenerational effect	Last till	Reference
Mice and human	BPA	miR-21, miR-146a and miR-200 family	Endometriosis, impair the ovarian granulose cell function	PTEN, it blocks PI3K/AKT signaling pathway	Possible endometriosis	N/A	[15]
Human stem cells	Cadmium	miR-92a-2–5p	Granulose cell apoptosis	Bcl-2, an anti-apoptotic gene	Granulose cell apoptosis	F3	[17]
Human	N/A	Increase in sperm miR-135b	Impaired reproductive capacity	Dmt1	Affect sperm development, viability	N/A	[26]
Humans (children)	N/A	MiR-92a-3p and miR-155–5p	Increased risk of obesity followed by GDM	Suppresses KLF2 and KLF4 to promote inflammation	Low pancreatic beta-cell adaptation to increased insulin demands	N/A	[43]
				Targets PPAR to disrupt insulin signaling			[30]
Mice and Humans	N/A	MiR-148a	Accelerates lipid accumulation and adipocyte differentiation	Represses Wnt1;Supports PPAR γ transcription	MiR-148a goes inside the exosomes to placenta	N/A	[32]
Mice	Vinclozolin	miR-23b, miR-21, let-7	Reduction in the PGCs number per testis, increased number of impairments in the seminiferous epithelium tubules and hypertonic cells, reduced ovarian reserve, premature ovarian insufficiency	BLIMP1, a transcription factor that is for specification of PGCs	Gonadal impairment	F2	[18]
Mice	Dexamethasone	miR-466b-3p	Loss of suppression of HDAC7, cause upregulation of HDAC7	Low expression of steroidogenic acute regulatory (StAR) protein and the 3β-HSD gene, leading to enlarged intra-testicular interstitial region and shrunken nucleus	Enlarged intra-testicular interstitial region and shrunken nucleus	F3	[19]
Mice	Silver nanoparticles	miR-465c-1 upregulated, miR-106a-5p downregulated	Production of abnormal sperm	Egr1	Sperm abnormalities, such as head and tail	F2	[25]
Mice	Dexamethasone	miR-17–5p in F1 upregulated	Decrease in the number of primordial follicles, induce diminished ovarian reserve	FOXL2/CDKN1B	Diminished ovarian reserve	F3	[23]
Mice	Tributyltin (TBT)	MiR-223	Diverts the mesenchymal stem cells toward the adipocyte lineage	Decreases expression of gene ZEB1, NFIB and FOXP1	Trans-generation obesity by promoting adipogenesis	F4	[36]
Mice	N/A	MiR-19b and miR-29a	Increase fasting glucose levels and impair glucose tolerance	Suppresses SIRT1 (supports pro-inflammation)ReducesG6PC(glucose intolerance)	Transmitted across generations through testes changes	F2	[39][42][40]
Mice	N/A	MiR-155 and miR-21	Regulates inflammation in obese mice	Silence genes that repress the production of proinflammatory TNF-α proteins	Paternal epigenetic inheritance through altered miRNA in sperm	N/A	[45]
Mice	N/A	MiR-34c and miR-449	Alleviates anxiety and defective sociability	CDK6, c-MYC, HDAC1 and BCL-2 expression reduced	F1 males inherited with reduced sperm miR-34/449, passing down to females (F2 and F3)	F3	[50]
Mice	N/A	MiR-375	Soma-to-Germline communication affecting miRNA expression in sperm	Mecp2, Cnr1, Crhr2, Prkcc regulation through miRNA-mediated methylation via DNA methyltransferase	Hippocampal miR-375 reached testes through circulation and altered miRNA expression in sperm	F3	[51]
Mice	Dexamethasone	MiR-466b-3p	Regulates testosterone synthesis	Impair acetylation of the StAR gene	Infertility and delayed puberty	F3	[19]
Rats	Cadmium	miR-124–3p, miR-133a-5p, miR-150–5p, let-7a-5p, miR-134–5p, and miR-30c-1–3p	Altered hormone levels (Estradiol,progesterone)	StAR, Cyp11a1, Cyp17a1, Cyp19a1, and Sf-1	Hormone levels altered, based on generation	F3	[23]
Fish	N/A	miRNA-525-DIAPH2, novel miRNA-525-MYOCD, and novel miRNA-525-RAI14	Reproductive impairment, dysregulation of ovaries	E.g. DIAPH2, for organization of actin cytoskeleton and ovarian function	Dysregulation of ovaries	F2	[20]
Fish	N/A	NC_007115.7.7_998413	Inhibit sperm flagella formation, lead to sperm malformation, and decreasing the male fertility	Spata2	Sperm malformation	N/A	[24]
Chicken	N/A	miR-301a-5p	Inhibit the regulation of TGFB2 transcript, affect spermatogenesis and sperm quality	TGFB2	Affect sperm quality	N/A	[23]

## Conflicts of interest

None declared.

## Supplementary Material

dvag035_Supplemental_Files
